# An experimental test of temperature‐dependent selection on mitochondrial haplotypes in *Callosobruchus maculatus* seed beetles

**DOI:** 10.1002/ece3.6775

**Published:** 2020-09-17

**Authors:** Elina Immonen, David Berger, Ahmed Sayadi, Johanna Liljestrand‐Rönn, Göran Arnqvist

**Affiliations:** ^1^ Department of Ecology and Evolution/Evolutionary Biology Uppsala University Uppsala Sweden; ^2^ Department of Ecology and Evolution/Animal Ecology Uppsala University Uppsala Sweden

**Keywords:** experimental evolution, genotype‐by‐environment interaction, mitochondrial DNA, mitonuclear epistasis, Mother's Curse, Pool‐seq, seed beetle, sex‐specific selection, temperature adaptation

## Abstract

Mitochondrial DNA (mtDNA) consists of few but vital maternally inherited genes that interact closely with nuclear genes to produce cellular energy. How important mtDNA polymorphism is for adaptation is still unclear. The assumption in population genetic studies is often that segregating mtDNA variation is selectively neutral. This contrasts with empirical observations of mtDNA haplotypes affecting fitness‐related traits and thermal sensitivity, and latitudinal clines in mtDNA haplotype frequencies. Here, we experimentally test whether ambient temperature affects selection on mtDNA variation, and whether such thermal effects are influenced by intergenomic epistasis due to interactions between mitochondrial and nuclear genes, using replicated experimental evolution in *Callosobruchus maculatus* seed beetle populations seeded with a mixture of different mtDNA haplotypes. We also test for sex‐specific consequences of mtDNA evolution on reproductive success, given that mtDNA mutations can have sexually antagonistic fitness effects. Our results demonstrate natural selection on mtDNA haplotypes, with some support for thermal environment influencing mtDNA evolution through mitonuclear epistasis. The changes in male and female reproductive fitness were both aligned with changes in mtDNA haplotype frequencies, suggesting that natural selection on mtDNA is sexually concordant in stressful thermal environments. We discuss the implications of our findings for the evolution of mtDNA.

## Graphical abstract

How important mtDNA polymorphism is for adaptation is still unclear. This study tests whether ambient temperature affects selection on mtDNA variation, and whether such thermal effects are influenced by intergenomic epistasis due to interactions between mitochondrial and nuclear genes, using replicated experimental evolution in *Callosobruchus maculatus* seed beetles. Our results demonstrate natural selection on mtDNA haplotypes, with some support for thermal environment influencing mtDNA evolution through mitonuclear epistasis. We also test for sex‐specific consequences of mtDNA evolution on reproductive success, given that mtDNA mutations can have sexually antagonistic fitness effects, but find that the changes in fitness were aligned in the sexes.

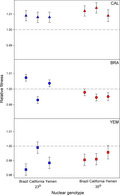

## 1 INTRODUCTION

Mitochondria have a central role in cellular energy metabolism regulated by enzyme complexes encoded by both mitochondrial and nuclear genes (Blier, Dufresne, & Burton, 2001; Rand, Haney, & Fry, 2004). The function of these complexes is highly sensitive to temperature (Ekstrom et al., 2017; Lemieux, Tardif, & Blier, 2010; Pichaud, Ballard, Tanguay, & Blier, 2013), which suggests that the genes could be involved in thermal adaptation. However, mitochondrial genes have traditionally been thought to evolve under strong purifying selection, due to their vital role in cellular respiration, with only selectively neutral mitochondrial mutations segregating as common alleles within and among populations (Ballard & Kreitman, 1994; Rand, 2001). These views have been questioned by several lines of evidence suggesting that polymorphisms in mitochondrial DNA (mtDNA) show signatures of recurrent adaptive evolution (Bazin, Glemin, & Galtier, 2006; Galtier, Nabholz, Glemin, & Hurst, 2009; James, Piganeau, & Eyre‐Walker, 2016) and contribute to variation in fitness‐related traits (Burton, Pereira, & Barreto, 2013; Dobler, Rogell, Budar, & Dowling, 2014; Dordevic et al., 2017; Dowling, Friberg, & Lindell, 2008; Immonen, Collet, Goenaga, & Arnqvist, 2016; Immonen, Ronn, Watson, Berger, & Arnqvist, 2016; Rand, Clark, & Kann, 2001; Wolff et al., 2016). Moreover, laboratory studies show that mtDNA polymorphism and epistasis between mitochondrial and nuclear genomes can affect expression of life‐history traits in a temperature‐dependent manner (Arnqvist et al., 2010; Dowling, Abiega, & Arnqvist, 2007; Hoekstra, Siddiq, & Montooth, 2013). Even the germ‐line rates of transmission of different mtDNA variants in heteroplasmy are sensitive to thermal conditions (Doi, Suzuki, & Matsuura, 1999). There is now also accumulating evidence that variation in mtDNA haplotype frequencies is associated with clinal variation in temperature (Quintela, Johansson, Kristjansson, Barreiro, & Laurila, 2014), altitude (Cheviron & Brumfield, 2009; Fontanillas, Depraz, Giorgi, & Perrin, 2005), or latitude (Camus, Wolff, Sgrò, & Dowling, 2017; Consuegra, John, Verspoor, & de Leaniz, 2015; Silva, Lima, Martel, & Castilho, 2014), including in humans (e.g., Mishmar et al., 2003). These studies have prompted the newly coined hypothesis of *mitochondrial climatic adaptation* (Camus et al., 2017), but direct experimental tests of the thermal environment driving changes in mtDNA haplotype frequencies are scarce (Lajbner, Pnini, Camus, Miller, & Dowling, 2018).

The female‐limited inheritance of mitochondria suggests that mtDNA effects may be sex‐specific (Frank & Hurst, 1996; Gemmell, Metcalf, & Allendorf, 2004), a prediction supported by several studies (e.g., Camus, Wolf, Morrow, & Dowling, 2015; Dobler et al., 2014; Immonen, Collet, et al., 2016; Innocenti, Morrow, & Dowling, 2011; Yee, Sutton, & Dowling, 2013). Female‐limited optimization of mitochondrial function could even lead to a genetic conflict with males (termed Mother's Curse; Frank & Hurst, 1996; Gemmell et al., 2004), if selection favors different mitochondrial haplotypes in the sexes. However, environmental stress is predicted to align selection in the sexes (e.g., Berger et al., 2014; Connallon, 2015; Long, Agrawal, & Rowe, 2012), which should lead to congruent fitness consequences of mtDNA evolution in the sexes. Studies examining the impact of mtDNA variation on each sex have typically been conducted under conditions to which organisms have been well adapted, which limits the ability to predict effects on sex‐specific fitness under environmental change.

Here, we experimentally test for selection on mtDNA haplotypes under two alternative stressful thermal regimes, using an approach combining replicated experimental evolution and sequencing of mtDNA using Pool‐Seq (Schlotterer, Tobler, Kofler, & Nolte, 2014) in laboratory populations of *Callosobruchus maculatus* seed beetles. We test for consistency of frequency changes of three mtDNA haplotypes (from known and equal starting frequencies) expressed in either of three genetically variable but divergent nuclear genetic backgrounds. Beetles were also phenotyped for sex‐specific lifetime reproductive success at two points in time. This design allowed us to test for fitness differences between our lines and whether these were associated with mtDNA haplotypes, mitonuclear epistasis, or thermal conditions. Previous work has demonstrated effects of mtDNA variation as well as mitonuclear epistasis on various reproductive and life‐history traits during different ontogenetic stages, under the thermal conditions to which beetles are well adapted (Arnqvist et al., 2010; Immonen, Collet, et al., 2016; Immonen, Ronn, et al., 2016). Under these conditions, female lifetime reproductive success is highest with a coevolved combination of nuclear and mitochondrial genes, whereas mtDNA effects are more idiosyncratic and age‐dependent in males (Immonen, Collet, et al., 2016). We therefore predicted that such non‐neutral effects may influence mtDNA evolution under stressful thermal conditions, with putatively sex‐specific fitness consequences.

## 2 MATERIALS AND METHODS

### 2.1 Species and rearing conditions

*Callosobruchus maculatus* is a cosmopolitan aphagous pest species of legume seeds, adapted to arid and warm conditions, and with a polygamous mating system. The standard laboratory rearing conditions for the mitochondrial introgression lines used for creating the laboratory evolution lines for this study were 29**°**C, a 12:12 light/dark cycle, and 50% relative humidity. We used black‐eyed beans (*Vigna unguiculata*) as a host species for both standard and experimental rearing.

Endosymbionts such as *Wolbachia* that could confound cytoplasmic genetic effects have been screened for in many *C. maculatus* populations, including the ones used here, using both dedicated PCR screens and deep short read sequencing of genomic DNA but have never been detected (see for example Tuda, Ronn, Buranapanichpan, Wasano, & Arnqvist, 2006). Nevertheless, all introgression lines used for creating the experimental evolution lines (see below) were treated with antibiotics to clear any cytoplasmic bacterial infections (Kazancioglu & Arnqvist, 2013).

### 2.2 Creation of the experimental evolution lines

To test whether thermal selection acts on mtDNA haplotype frequencies, we exposed experimental beetle lines (described below) to either a warmer, 35**°**C, or a colder, 23**°**C, thermal environment, for 36 and 23 generations, respectively. These two conditions are both stressful compared to the optimal temperature of 29**°**C to which the beetles are adapted (Martinossi‐Allibert, Arnqvist, & Berger, 2017; Stillwell, Wallin, Hitchcock, & Fox, 2007).

To create the experimental evolution populations, we used previously created lines in which three mitochondrial haplotypes (BRA, CAL, and YEM) have been introgressed in an orthogonal fashion into three outbred nuclear genetic backgrounds (*Brazil, California*, and *Yemen*) through repeated backcrossing for over 16 generations (Arnqvist et al., 2010; Dowling, Abiega, & Arnqvist, 2007; Dowling, Friberg, & Arnqvist, 2007; Dowling, Meerupati, & Arnqvist, 2010; Dowling, Nowostawski, & Arnqvist, 2007; Immonen, Collet, et al., 2016; Immonen, Ronn, et al., 2016; Kazancioglu & Arnqvist, 2013). These haplotypes were captured from allopatric populations, but according to previous work, they all belong to haplotype groups that segregate sympatrically in West African populations of *C. maculatus* (Kebe et al., 2016). We further tested this by directly comparing the three experimental haplotypes to 43 lines sampled from populations within West Africa (see Appendix S1 for the methods, results and Figure S1). The three experimental mtDNA haplotypes were selected out of a larger set of haplotypes known to carry nonsynonymous substitutions (Arnqvist et al., 2010). The resulting nine mitonuclear introgression lines were created in duplicates (a total of 18 lines), starting from two different females (i.e., mitochondrial Eves) for each haplotype (Figure S2). The lines were then kept as separate populations for approximately 50 generations. Before the start of our experiment, we performed (consecutively) two additional generations (17th and 18th) of backcrossing of females from each introgression line to outbred males from their nuclear background source population, to minimize any putative effects of line‐specific mitonuclear coadaptation that could have occurred since the creation of the lines. We did this by randomly sampling five females from each introgression line and mating them individually to five males from each of the source populations harboring the nuclear genetic background corresponding to that of the female. F1 daughters (*N* = 20 from each pair) were again backcrossed with males (*N* = 20) from the nuclear background source population. We verified the correct mtDNA haplotype of each line with a standard diagnostic PCR (see Kazancioglu & Arnqvist, 2013 for PCR conditions and primers).

We used these mitonuclear introgression lines to create 24 experimental evolution populations in which all three mtDNA haplotypes sharing the same nuclear genetic background were introduced in exactly equal proportions (1/3). This resulted in three types of mtDNA‐mix lines, only differing in the nuclear genotype. To do this, we seeded each of the 24 populations with 50 females of each of the three mtDNA haplotypes. These 150 females, all of which shared their nuclear genotype, were placed together with 150 males with the same nuclear genotype as the females. The eight mtDNA‐mix replicates for each of the three nuclear genotype represent two biological replicates (mitochondrial Eves), two technical replicates, and the two thermal environments (35**°**C and 23**°**C) (2 × 2 × 2). One of the four replicates of mtDNA‐mix with a *Yemen* nuclear background evolving under 35**°**C was lost during culturing. As a control to the mtDNA‐mix lines used in the reproductive performance assays (see below), we simultaneously initiated lines with a single mtDNA haplotype native to the given nuclear genome (150 females and males). These pure‐mtDNA lines were used in the reproductive fitness assays (see below), and were also subjected to the same thermal regimes, and replicated in the same fashion (i.e., 2 × 2 × 2). Figure S2 provides a summary of the experimental scheme used. The lines were cultured in 1 L glass jars with 200 ml of *V. unguiculata* and a population size of approximately 600 adults at the start of each generation. The generation times for the lines evolving under 35**°**C and 23**°**C were approx. 21 and 35 days, respectively.

### 2.3 Sample preparation, DNA extraction, and sequencing

We assessed the evolution of mitochondrial haplotypes by estimating their frequency changes from the initial 1/3 using a pool‐seq protocol (Schlotterer et al., 2014). Sequencing pools of individuals allows the estimation of relative haplotype frequencies by using read count information. Assigning reads to either of the three haplotypes was made possible by recent mitogenome assemblies of these three mitogenomes (Sayadi, Immonen, Tellgren‐Roth, & Arnqvist, 2017), which we used to identify polymorphic sites and to map reads (see below). Pool‐seq strategies can be sensitive to variation in allele frequencies due to random sampling bias (Ferretti, Ramos‐Onsins, & Perez‐Enciso, 2013). To minimize such error, we sequenced pools of 100 individuals (Schlotterer et al., 2014).

We sequenced one sample from each of our 23 mtDNA‐mix lines. For each sample, we randomly selected 100 adult male beetles and extracted high‐quality DNA using a salt–ethanol precipitation protocol (see Appendix S1: Methods). We also sequenced three independent control samples, to validate our methods. In each of these samples, we pooled 33 males from pure lines known to carry each of the three mtDNA haplotypes and then extracted DNA as above from the 99 males (3 × 33) per sample. Here, exact haplotype frequencies in the samples were thus known (i.e., 1/3).

Sequencing libraries were prepared using the TruSeq PCRfree DNA library preparation kit. The resulting 26 libraries were then subjected to cluster generation and 125 cycles paired‐end sequencing in 4 lanes using the HiSeq2500 system and v4 sequencing chemistry. In total, we sequenced on average 41 million reads per sample. These were then mapped to the three mitogenomes using Bowtie (Langmead & Salzberg, 2012) (with parameters ‐a ‐‐best –strata ‐v) and BWA‐MEM with default parameters (Li & Durbin, 2010). Allowing 0–3 mismatches, an average of 51k reads per sample (*SD* = 15k, Range = 28k–79k) mapped uniquely to either one of the three mitogenomes yielding an effective mean mitogenome coverage of 255× (this is not including uninformative reads that mapped to more than one mitogenome). Allowing for 0 mismatches, the corresponding numbers were 12k reads per sample (*SD* = 4k, Range = 7k–19k) and an effective coverage of 60×. Here, we used read count data only from reads that mapped to a single mitogenome with 0 mismatches.

### 2.4 Estimation of mtDNA haplotype frequency changes

In our three control samples, the proportion of total reads that mapped to each of the three different mtDNA haplotypes ranged between 30.7%–35.1%, 30.1%–38.1%, and 32.3%–35.0%, respectively. For each of the 13 protein‐coding mtDNA genes (PCGs), we assessed potential procedural bias by testing whether the observed number of reads that mapped to a given haplotype differed significantly from the expected (i.e., 1/3) in each control sample, using contingency table chi‐square tests. This showed that read counts for seven PCGs did not deviate significantly (FDR; *Q* > 0.05) from the expectation in either of the three control samples. Rather than correcting for potential bias in some PCGs, we used only read counts for the seven PCGs that showed no biases (*cox3, nad3, nad4l, cox2, atp8, nad5, and nad1*) to assess mtDNA haplotype frequencies in our mtDNA‐mix lines. For each of the seven PCGs, we used the number of reads mapping to a particular haplotype divided by the sum of reads mapping to either of the three haplotypes in a given sample as an estimate of the proportional representation of that haplotype in that sample. The estimates of haplotype frequency based on different PCGs were highly consistent (mean correlation across lines: *r* = .91). Here, we used the mean proportion over the seven PCGs as our measure of haplotype frequency in a given sample.

For each evolving population, we calculated the relative fitness of a given haplotype, *W_i_*, from

(1) $$p_{i}\left(t+1\right)=\frac{p_{i}\left(t\right)W_{i}}{\left(p_{\text{BRA}}\left(t\right)W_{\text{BRA}}+p_{\text{CAL}}\left(t\right)W_{\text{CAL}}+p_{\text{YEM}}\left(t\right)W_{\text{YEM}}\right)}$$

where *pi* is the frequency of the focal haplotype (*i* = BRA, CAL, and YEM), and *t* is the time of the start of the experiment. Relative fitness was then used as a response variable in our models of the effects of thermal regimes and nuclear genotype. The per‐generation haplotype frequency change averaged over the course of the experiment was estimated simply as Δ*f* = (*f_t_* – 1/3)/*t*, where *f_t_* is the observed proportion of a given haplotype in a sample and *t* is the number of generations between the start of our experimental evolution and when the sample was taken.

The unit of replication in experimental evolution studies is the evolving line (Kawecki et al., 2012), and this is reflected in our analyses. We note that we track frequency dynamics in replicated and independent lines and the nonfocal effect of random genetic drift, which is predicted to be considerable for mtDNA dynamics, forms a part of the residual term of our inferential models. First, we asked whether the relative fitness of the three haplotypes differed overall, inferred from consistent changes in the haplotype frequencies across the replicate populations. Second, we tested whether thermal regime and nuclear background affected the relative fitness of haplotypes. This involved fitting models for each haplotype separately, where thermal regime, nuclear background, and their interaction were fixed effects. Needless to say, these three models are not independent. An omnibus test of whether thermal regime and nuclear background affected the relative fitness of all haplotypes is complicated by the fact that the response matrix is based on compositional data and suffers from the zero‐sum constraint, as the three haplotypes’ relative fitness sum to one. To allow modeling all aspects of haplotype frequency changes in our experiment, we thus first reduced the dimensionality of our response matrix from three collinear dimensions to two orthogonal dimensions by means of a standard principal component analysis, based on the correlation matrix. The resulting two derived variables (*W*_PC1_ and *W*_PC2_) collectively describe 100% of the variance in relative fitness of the three haplotypes across our samples. We then fitted a repeated‐measures ANOVA where the two aspects of variation in relative fitness (*W*_PC1_ and *W*_PC2_) were the nonfocal within‐subjects factor with two levels, while thermal regime, nuclear background, and their interaction were focal fixed effects between‐subjects factors. This represents our main inferential model. Here, we also tested for the effects of biological replicate (i.e., mitochondrial Eve), but this factor was dropped as it had no significant effects. We note here that the latter fact is unsurprising, given that the two mitochondrial Eves from each population have been shown to share the same mtDNA haplotype (Sayadi et al., 2017).

### 2.5 Estimation of genetic differences

Genetic differences in the mitochondrial genes between the three mitochondrial haplotypes (GenBank accession numbers KY942060, KY942061, and KY942062; Sayadi et al., 2017) were assessed using *Geneious* (Kearse et al., 2012). Nucleotide diversities and the haplotype network analysis with neighbor‐joining network were conducted using Jalview (Waterhouse, Procter, Martin, Clamp, & Barton, 2009) We used *MudPred2* to test the probability that the amino acid variants between haplotypes cause pathogenic protein structure and function (Li et al., 2009).

### 2.6 Lifetime reproductive success (LRS)

We tested the effect of mitochondrial genetic variation on the lifetime reproductive success (the lifetime number of fertile eggs) of male and female beetles, under competitive context, early (3rd generation) and after 33 generations of evolution under novel thermal stress. We had two motivations for these assays. First, we wanted to assess whether the relative reproductive fitness differences between the mtDNA haplotypes (relative LRS) evolved in concert with mtDNA haplotype frequencies, and could thus explain how selection on mtDNA may arise. Second, we wanted to test for sex differences in these effects. Due to logistical constraints, we were able to focus only on one of the temperature regimes and chose the warmer temperature (35**°**C). In assays of female LRS, we placed two 24 hr‐old virgin females from each of the mtDNA‐mix and pure lines (i.e., with the same nuclear background but no mtDNA variation) with two standard males that shared the same nuclear background. We calculated the total number of fertile eggs the females produced during their lifetime. In male assays, for each mitochondrial‐mix and pure line, we housed a single male together with a sterilized standard competitor male and two standard females (with the same nuclear genetic background as the focal male). The competitor males were sterilized by ionizing irradiation (with 100 Gy; Grieshop, Stangberg, Martinossi‐Allibert, Arnqvist, & Berger, 2016). This procedure has no detectable effects on male mating success or female fecundity, and is therefore a standard practice for obtaining estimates of competitive reproductive fitness for this species (Berger et al., 2014; Grieshop & Arnqvist, 2018; Grieshop et al., 2016). We counted the lifetime number of fertile eggs, which thus were fertilized by the focal male. The female and male assays were replicated a total of 296, 303, and 259 times for the mtDNA‐mix lines and 293, 303, and 271 times for the pure lines with *Brazil*, *California,* and *Yemen* nuclear backgrounds, respectively, across the two generation points and sexes, with an average of 19 assays per sex, time point and replicate line (total *N* of the experiment = 1,725). As the number of contributing individuals differed in the female and male assays, we divided the number of eggs by two in the female assays to make the datasets comparable in our analyses.

We used two analytical approaches to meet our goals. First, we used a random regression model (fitted using restricted maximum likelihood) with the package *lme4* (Bates, Maechler, Bolker, & Walker, 2014) in R (v.3.2.2; R Development Core Team, 2011), with log‐normalized lifetime number of eggs as the response variable. We fitted sex, line type (mtDNA‐mix or pure), nuclear genetic background and generation (representing the effect of selection), and their 2‐ and 3‐way interactions as fixed effect factors. To account for the replicate structure and line‐specific generation and sex effects, we included the following random effect terms: line replicate, replicate:sex, replicate:generation, and replicate:generation:sex. By fitting the line (with 23 levels) as a random factor, it becomes implicitly nested within the fixed effect factors, ensuring a correct data structure and a proper estimation of degrees of freedom. The significance of terms in the final model was assessed with ANOVA type III (*F* tests with Kenward–Rogers estimation of the degrees of freedom) using the *car* package (Fox & Weisberg, 2011).

The “pure” lines with a single mtDNA haplotype represent controls for each mtDNA‐mix line that share the same nuclear genotype (Figure S2), and provide information on the relative reproductive fitness of each haplotype in their native nuclear genetic backgrounds. Any difference over time between the mix and pure line types in a given nuclear background should thus reflect the mtDNA genetic effects (whether additive or epistatic), signified as an interaction between line type (mtDNA‐mix or pure), nuclear background and generation. We also predicted that if fitness consequences of changes in mtDNA frequencies over time are sex‐specific, we should see this as an interaction effect between sex, generation, and line type. We also hypothesized that because male adaptation can only evolve through nuclear genetic variation, the nuclear genetic component may show more sex‐specificity over time, signified by an interaction effect between nuclear background, sex, and generation.

Our second analytical approach tested more directly for an association between variance in sex‐specific reproductive success and the observed haplotype frequency changes across lines. The analyses of the haplotype frequency data showed that, overall, the relative fitness rank of mtDNA haplotypes was CAL > BRA > YEM. We thus hypothesized that, among our mtDNA‐mix lines, the phenotypic evolution of relative reproductive success during our experimental evolution should to some extent reflect the evolution of haplotype frequencies in a given line. Here, the prediction is that lines in which the increase in CAL haplotype frequency was relatively high should also show a higher increase in relative LRS, while the opposite should hold true for the YEM haplotype. Although our statistical power to detect such associations was limited, given only 11 evolving lines, we tested this prediction using the following rationale.

We first estimated average sex‐specific relative LRS, as a proxy for fitness (*W*), of each mix line at generation 3 and 33, by dividing average sex‐ and generation‐specific LRS with the average sex‐ and generation‐specific LRS of the CAL pure line. This measure of fitness thus expresses the fitness of a given mix line, relative to the genotype that should show highest fitness (CAL pure). For each sex, we then subtracted relative fitness at generation 3 from that at generation 33 to attain the change in fitness of a given line as

$$\Delta W=W_{33}‐W_{3}$$

This expresses the change in sex‐specific fitness, relative to the most fit genotype, over the course of our experimental evolution. To relate Δ*W* to Δ*f* of the three mtDNA haplotypes, given that the three measures of Δ*f* represent compositional data and suffer from the zero‐sum constraint, we used partial least squares (PLS) modeling. Here, the two measures of Δ*W* (male and female) were treated as response variables and the Δ*f's* of the three mtDNA haplotypes as predictors. Inferences were restricted to the first dimension. The PLS model was evaluated by bootstrapping (10k bootstrap replicates), using bias corrected confidence intervals following correction for axis reversals (Hair, Hult, Ringle, & Sarstedt, 2017) in SmartPLS v. 3.2.7 (Ringle, Wende, & Becker, 2015).

## 3 RESULTS

### 3.1 Haplotype frequency changes

**FIGURE 1:**
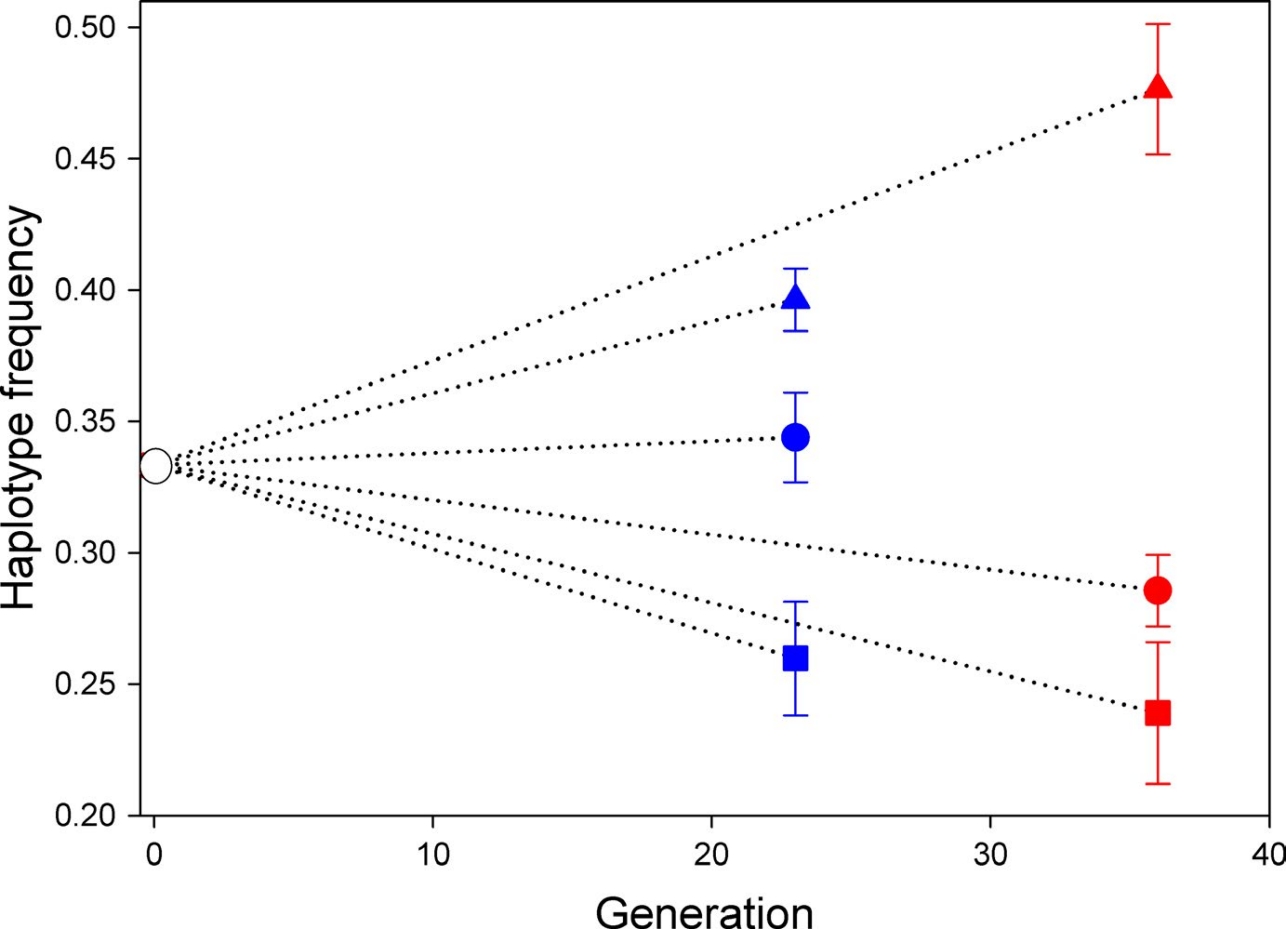
Overall mtDNA haplotype frequency changes during experimental evolution under cold (23°C; blue symbols) and hot (35°C; red symbols) conditions. All populations were started with a frequency of 0.33 for each of the three haplotypes at generation 1. CAL haplotype (triangles) increased overall in frequency, the YEM haplotype (squares) decreased while the BRA haplotype (circles) changed less. Shown are means ± *SE* haplotype frequencies at the end of the experiment

The three haplotypes (Figure 1) differed significantly in relative fitness (*F*_2,20_ = 39.2, *p* < .001), with CAL showing the highest overall relative fitness (mean *W*; 95% bias corrected bootstrap CI (9,999 bootstraps): 1.010; 1.007–1.0125), followed by BRA (0.999; 0.996–1.002) and YEM (0.991; 0.988–0.995). Pairwise between‐haplotype comparisons of mean *W* confirmed this pattern (Tukey's post hoc tests; CAL versus BRA: *p* < .001; CAL versus YEM: *p* < .001; BRA versus YEM: *p* = .002). The mean relative fitness of CAL (*H*_0_: *W* = 1; *t* = 7.64, *p* < .001) and YEM (*t* = −4.90, *p* < .001) differed significantly from unity, but not that of BRA (*t* = −0–89, *p* = .385). This provides experimental evidence for natural selection on mtDNA.

**FIGURE 2:**
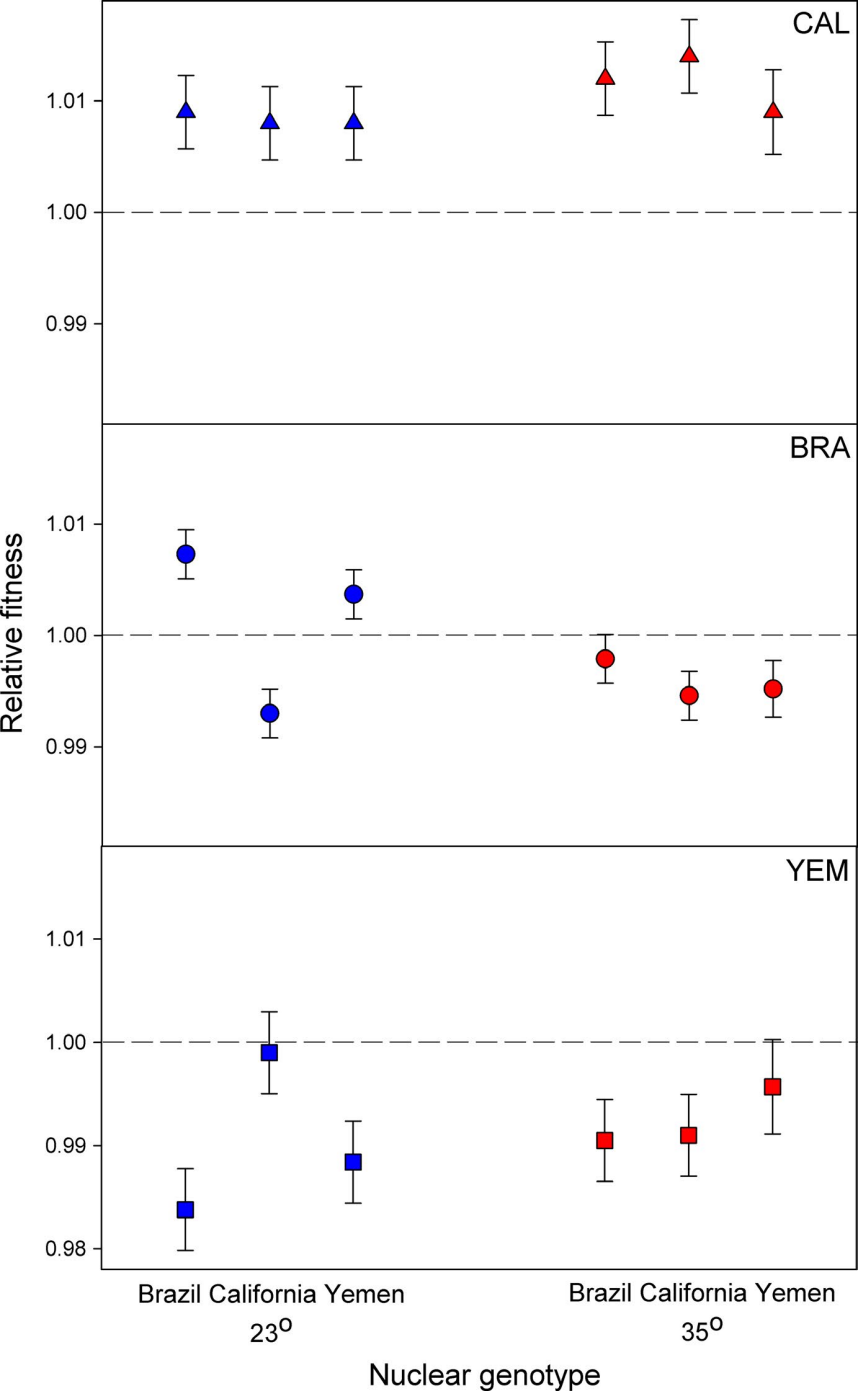
Mitonuclear epistasis for relative fitness depends upon haplotype and temperature. The CAL haplotype (triangles) showed high relative fitness in all nuclear backgrounds and temperatures, while the relative fitness of the BRA and the YEM haplotypes depended upon the nuclear genetic background and temperature regime. Shown are mean (± *SE*) relative fitness

Importantly, thermal regime and nuclear background both significantly affected selection on mtDNA haplotypes during our experiment (Table 1). The CAL and YEM haplotype showed a higher relative fitness at 35**°**C than at 23**°**C, while the opposite was true for the BRA haplotype (Figure 2). Moreover, the effect of thermal regime was to some extent contingent upon the nuclear genetic background (Tables 1 and 2, Figure 2). At 35**°**C, all three haplotypes showed highest relative fitness when expressed in their native nuclear background, consistent with mitonuclear coadaptation, but at 23**°**C, this was only true for the BRA haplotype. Further, at this colder temperature, BRA and YEM haplotypes expressed in the *California* nuclear background showed opposing fitness effects. Thus, the fact that the interaction between the nuclear genetic background and the thermal regime differed across mtDNA haplotypes (Table 2, Figure 2) provides evidence for a G × G × E interaction for relative fitness of haplotypes. This illustrates that mitonuclear epistasis can be contingent on temperature. See Table S1 and Figure S3 for data separately on each replicate line.

**TABLE 1:** The between‐line effects of temperature and nuclear genetic background on the relative fitness of all mtDNA haplotypes during experimental evolution, from a repeated‐measures ANOVA

Source	*df*	MS	*F*	*p*	*p*_rand_
Thermal regime	1	4.75	6.27	.023	.025
Nuclear background	2	5.23	7.55	.004	.006
Thermal × Nuclear	2	2.90	4.10	.035	.036
Error	17	0.71			

Note

Given are also nonparametric *p*‐values (*p*_rand_) from a permutation test of the model (9,999 random permutations).

**TABLE 2:** The effects of temperature and nuclear genetic background on the relative fitness of the three mtDNA haplotypes (BRA, CAL, and YEM) during the experimental evolution

Source	*df*	BRA			CAL				YEM		
		MS	*F*	*p*	MS	*F*		*p*	MS	*F*	*p*
Thermal regime	1	1.66E−04	8.59	.009	7.73E−05	1.78	.200		1.67E−05	0.27	.612
Nuclear	2	1.62E−04	8.38	.003	1.45E−05	0.33	.721		1.24E−04	1.97	.170
Thermal × Nuclear	2	7.41E−05	3.83	.042	1.32E−05	0.30	.743		1.45E−04	2.32	.128
Error	17	1.93E−05			4.34E−05				6.28E−05		

### 3.2 Lifetime reproductive success at a warmer temperature

The mtDNA haplotype frequency changes demonstrated selection overall favoring the CAL haplotype, although the magnitude of this relative fitness advantage was contingent upon thermal regime and nuclear background. We hypothesized that the relative fitness advantage could arise from variation in adult female reproductive success, potentially in a sex‐dependent manner. To test these, we first used a random regression model to compare the reproductive success of the lines harboring a mix of mtDNA haplotypes to those with a single mtDNA haplotype (i.e., mix and “pure” lines, Figure S2), in the different nuclear genetic backgrounds, early and after 33 generations of experimental evolution. Any difference over time in the reproductive performance between the populations with and without mtDNA variation in a given nuclear genetic background must arise due to a change in mtDNA haplotype frequencies, signified by an interaction effect between line type, nuclear background, and generation. We found only marginal support for this (*p* = .0802, Table S2).

We then used PLS modeling to assess associations between sex‐specific change in relative reproductive fitness (Δ*W*) and the observed pattern of mtDNA haplotype frequency changes across the experimental populations. The first PLS dimension explained approximately 37% (95% CI: 6%–83%) of the covariation between the changes in haplotype frequency and lifetime reproductive success. Inspections of the loadings on the first pair of latent variables (Table 3), which were positively related, showed that both male and female Δ*W* loaded positively on the latent variable, although only male Δ*W* significantly so. For the latent variable describing change in haplotype frequency, Δ*f* for CAL loaded significantly positively and Δ*f* for YEM significantly negatively. Hence, the pattern of covariation between phenotypic evolution of relative fitness and the evolution of haplotype frequencies during our experimental evolution conformed well with our predictions: An increase in the relative frequency of the CAL haplotype was associated with elevated reproductive fitness during the experiment, while increases in the relative frequency of the YEM haplotype were associated with a reduction in fitness. This is consistent with the hypothesis that the evolution of haplotype frequencies was associated with the evolution of relative reproductive success during our experimental evolution.

**TABLE 3:** Loadings on the two latent variables forming the first PLS dimension, describing covariation between mtDNA haplotype frequency changes and the evolution of relative sex‐specific fitness across replicated experimental evolution lines

		Loadings (W)	SE_W_	*t*	*p*	95% CI_W_
X‐set	Δ*f*_CAL_	0.591	0.216	2.74	.006	0.448 to 1.677
	Δ*f*_BRA_	−0.194	0.421	0.46	.644	−0.948 to 0.469
	Δ*f*_YEM_	−0.447	0.151	2.95	.003	−0.620 to −0.127
Y‐set	Δ*W*_male_	0.895	0.309	2.90	.004	0.533 to 1.459
	Δ*W*_female_	0.228	0.413	0.55	.580	−1.112 to 0.643

In contrast, we found no direct support for the hypothesis that evolution of mtDNA haplotype frequencies should affect the sexes differently (Figure S4). The random regression model showed no significant interaction effect of sex, generation, and line type on LRS, and there were also no significant differences between the sexes depending on the nuclear genetic background or the generation that was assayed (Table S2). In accordance, both sexes showed a positive loading on the latent variable describing covariation between mtDNA haplotype frequency changes and the evolution of relative reproductive fitness across the lines, and this loading did not differ significantly between the sexes (Table 3). Thus, thermal selection on mtDNA was overall sexually concordant during experimental evolution.

### 3.3 Molecular divergence across haplotypes

Analysis of the genetic differences in the mitochondrial genes between the three mitochondrial strains revealed a total of 180 single nucleotide polymorphisms (SNPs) (Figure S5) and one deletion of a single base pair. 166 of the SNPs were in the protein‐coding genes (PCGs), with 149 synonymous and 17 nonsynonymous substitutions (Tables S2 and S3). The YEM haplotype was overall more diverged from the other two (see Figure S3 for haplotype network). The pairwise nucleotide diversities (*π*) between BRA/CA, BRA/YEM, and YEM/CA haplotypes were 0.0047, 0.0101, and 0.0097, respectively. 63% of the PCG SNPs (total of 105) separated the YEM haplotype from the other two, while 20% (33 SNPs) were unique to BRA and 17% (28 SNPs) to CAL haplotypes (Table S3). Among the nonsynonymous substitutions, there were three amino acid changes unique to the CAL haplotype in the PCGs *nad2*, *cox1*, and *nad5*, while seven separated the BRA haplotype (in PCGs *cox2*, *nad5*, *nad4*, and *cob*) and six the YEM haplotype (in PCGs *nad2*, *cox3*, *nad4*, *cob*, and *nad1*) from the other two (Table S4) (see Appendix S1 for further results).

The role of mtDNA variation in thermal adaptation could in theory be amplified in our experiments if the three mtDNA haplotypes used, deriving from different populations, are much more divergent than haplotypes typically occurring sympatrically within populations of *C. maculatus*. A previous global analysis of this species (Kebe et al., 2016) showed that this is unlikely: Total genetic variance in mtDNA is dominated by within‐population (38.6%) and among‐populations‐within‐region (48.7%) variance, and among‐continent variance accounts for a much smaller fraction of total genetic variance (12.7%). Nevertheless, we assayed sympatric haplotype diversity in a natural population of *C. maculatus* to compare to the diversity of our three experimental haplotypes. The synonymous within‐population *π* was 0.041 and thus considerably higher than between any of the three experimental populations. The relationship among the sympatric and allopatric haplotypes (Figure S1) showed that the three experimental haplotypes are distinct, but are overall no more divergent than are haplotypes segregating sympatrically within the sampled population (see Appendix S1 for further information).

## 4 DISCUSSION

Our results demonstrate that mtDNA haplotype frequency dynamics are nonrandom and that they are contingent upon thermal conditions, providing experimental evidence against the widely held assumption of neutrality of segregating mitochondrial genetic variation. We also show that mtDNA haplotype frequency changes during experimental evolution are associated with changes in lifetime reproductive success across lines. Furthermore, we provide novel type of evidence for evolution of mtDNA haplotypes mediated by thermal selection depending upon the nuclear genetic background.

Although the overall relative fitness rank of particular haplotypes did not change across the two specific temperatures used during our experiment, the analysis of frequency changes showed a significant influence of genotype‐by‐genotype‐by‐environment interactions (Arnqvist et al., 2010). It is interesting to note that the relative fitness of the mitonuclear genotypes observed here under thermal stress was different compared to what we have previous observed under benign thermal conditions (Immonen, Collet, et al., 2016), which demonstrated epistasis for reproductive fitness as a result of mitonuclear co‐evolution (Immonen, Collet, et al., 2016). Here, we instead found that selection in stressful thermal environments consistently favored one of the haplotypes, irrespective of the nuclear genetic background, while the mitonuclear epistatic effects for the other two haplotypes were temperature dependent. Overall, the epistatic effects were more pronounced at the cold temperature, and the co‐evolutionary history of the two genomes played a role only in the warmer environment (Figure 2). Collectively, these observations suggest that selection can apparently act on mitochondrial‐nuclear combinations in a rather idiosyncratic fashion depending on the thermal environment.

When such G × G × E interactions occur in the wild, fluctuating thermal conditions over time and space could act to maintain variation in both mitochondrial genes and in the nuclear genes they interact with, and create transient distributions of mtDNA haplotypes (Arnqvist et al., 2010). This is expected because the mitochondrial function depends closely on the interaction with products from over a thousand genes encoded by the nuclear genome (Rand et al., 2004). In accordance, many studies have demonstrated mitonuclear epistasis in fitness‐related traits (Burton & Barreto, 2012; Dobler et al., 2014; Dowling, Abiega, & Arnqvist, 2007; Dowling, Friberg, Hailer, & Arnqvist, 2007; Dowling et al., 2010; Ellison & Burton, 2008; Immonen, Ronn, et al., 2016; James & Ballard, 2003; Meiklejohn et al., 2013; Rand et al., 2001; Rand, Fry, & Sheldahl, 2006; Yee, Rogell, Lemos, & Dowling, 2015).

However, selection favored the CAL mtDNA overall, which suggests that local conditions could also fix mitochondrial variation due to beneficial mutations. The relative success of the CAL haplotype could also be explained by the fact that it carries the fewest (putatively deleterious) SNPs of the three haplotypes. Interestingly, the advantage of the CAL haplotype was greatest at 35°C, broadly in agreement with recent theory (Agozzino & Dill, 2018; Berger, Stångberg, Baur, & Walters, 2018) and empirical evidence (Berger et al., 2018; Dandage et al., 2018) suggesting that purifying selection on protein‐coding genes may be overall stronger at warm temperatures. We also note that additional negative frequency‐dependent selection could nevertheless act to maintain mtDNA variation (Camus et al., 2017; James et al., 2016; Kazancioglu & Arnqvist, 2013; Kurbalija Novicic, Sayadi, Jelic, & Arnqvist, 2020).

We assessed if the evolution of mtDNA haplotype frequencies results from variation in reproductive success and found some support for this. The relative fitness of haplotypes, based on their frequency evolution, was positively associated with variation in lifetime reproductive success across lines. However, the association was significant only in males, thus providing no direct support for adult female reproductive success explaining the increase in the CAL haplotype frequency under both temperature regimes. This weaker association in females suggests that selection has acted on mtDNA also during development, via juvenile survival. Indeed, the warmer temperature regime used here causes strong selection on juvenile survival relative to a more benign temperature in both sexes in *C. maculatus* (Martinossi‐Allibert et al., 2017). Mitochondrial polymorphism affects, for example, juvenile metabolic rate in this species in a temperature‐specific manner (Arnqvist et al., 2010), and it is thus likely that the net selection on mtDNA arises from both adult reproductive success and juvenile survival.

The mtDNA evolution had a similar association with the evolution of reproductive success in both sexes, despite previous work showing that mtDNA effects under benign conditions are highly dependent on the sex and even sexually antagonistic for some mitonuclear genotypes (Immonen, Collet, et al., 2016). This suggests that the fitness consequences of variation in mitochondrial function become more sexually concordant under thermal stress. Selection is generally predicted to be more aligned between the sexes under novel or harsh environmental conditions (Long et al., 2012; Berger et al., 2014; Connallon, 2015; but see Martinossi‐Allibert et al., 2018), limiting the scope for a male‐specific genetic load from mtDNA mutations. Further work across species and a range of temperatures is needed to test whether Mother's curse effects are generally reduced during adaptation to stressful conditions.

The fact that mtDNA harbors non‐neutral variation segregating within and among populations has now been demonstrated across several taxa (Ballard & Rand, 2005; Dowling et al., 2008; Rand, 2001). Despite this, studies continue to use mitochondrial substitutions as markers of neutral population divergence in phylogenetic and phylogeographic studies. Our results strengthen the caution against such practice (Dowling et al., 2008; Galtier et al., 2009; William, Ballard, & Kreitman, 1995). We find, for example, that among our three experimental mtDNA haplotypes, there are 17 nonsynonymous substitutions in the protein‐coding genes, with three unique to the CAL haplotype (in the genes *nad2, cox1,* and *nad5*) which was favored by selection. A recent study estimated that up to 60% of nonsynonymous substitutions in insect mitochondria have undergone adaptive evolution across species (James et al., 2016). Rather than demographic processes, it is therefore likely that mtDNA diversity often reflects patterns of selective sweeps (James et al., 2016), or balancing selection (Kurbalija Novicic et al., 2020), and our results suggest that thermal selection can also be important in this process.

In summary, while our results do not support a major role of mtDNA in thermal adaptation per se, they provide strong evidence against neutrality of mtDNA and suggest that the thermal environment plays a role in affecting mtDNA evolution via epistatic mitonuclear interactions. Finally, the finding that the reproductive fitness effects of mtDNA frequency evolution were aligned in the sexes suggests that thermal stress may generate sexually concordant selection on mtDNA.

## CONFLICT OF INTEREST

All authors declare that they are free of competing interests.

## AUTHOR CONTRIBUTION

**Elina Immonen:** Conceptualization (supporting); Data curation (equal); Formal analysis (equal); Investigation (equal); Methodology (equal); Project administration (lead); Visualization (equal); Writing‐original draft (lead); Writing‐review & editing (lead). **David Berger:** Conceptualization (equal); Formal analysis (supporting); Investigation (supporting); Methodology (supporting); Project administration (supporting); Visualization (supporting); Writing‐review & editing (supporting). **Ahmed Sayadi:** Data curation (equal); Formal analysis (equal); Methodology (supporting). **Johanna Liljestrand‐Rönn:** Methodology (supporting); Resources (supporting). **Göran Arnqvist:** Conceptualization (equal); Formal analysis (equal); Funding acquisition (lead); Methodology (equal); Supervision (lead); Visualization (equal); Writing‐original draft (supporting); Writing‐review & editing (supporting).

## Supporting information

Appendix S1

Table S1
