# Supplementary material for: An experimental test of temperature‐dependent selection on mitochondrial haplotypes in Callosobruchus maculatus seed beetles
Source: Ecol Evol. 2020 Sep 17;10(20):11387–98. doi: 10.1002/ece3.6775 (PMC7593184; doi:10.1002/ece3.6775)
Supplement: Supplementary file 1 — Appendix S1 [file ECE3-10-11387-s001.pdf]

Experimental test of natural selection from temperature stress on mitochondrial haplotypes in *Callosobruchus maculatus* seed beetles

Supplementary Material

Content:

Supplementary Methods
Supplementary Results

Tables S2-4
Figures S1-5

Obs! Supplementary table S1 provided as a separate file (TableS1_Data_IndividualReplicates.xlsx)

Supplementary Methods

DNA extraction protocol

For each sample, we randomly selected 100 adult male beetles and extracted high-quality DNA using a salt-ethanol precipitation protocol. Beetles were first gently macerated and placed in preparation buffer (100 mM NaCl, 10 mM Tris-HCl, pH = 8.0, 0.5% SDS) together with proteinase K, vortexed and incubated at 50°C overnight. Samples were then frozen overnight. To precipitate DNA, we added saturated NaCl several times before adding 95% ethanol, and then spun the DNA into a pellet. The DNA pellet was suspended in TE buffer (pH = 7.6). DNA quality and quantity were assessed using NanoDrop, Qubit and Bioanalyzer.

Supplementary Results

Molecular divergence across haplotypes

Using the *MutPred2* (Li et al. 2009) algorithm we assessed putative pathogenicity of the nonsynonymous substitutions found between the three mtDNA haplotypes. This analysis identified three SNPs across two genes as potentially pathogenic: in *cob* a change from V to I (in BRA, at a position 296) alters the transmembrane protein (probability score = 0.25, $P < 0.0001$, affected ELM motifs: ELME000333, 000336), and a change from N to S (in YEM, at a position 173. Affected ELM and PROSITE motifs: ELME000053, ELME000070, ELME000085, ELME000239, PS00001) causes altered ordered interface (prob. score = 0.25, $P = 0.02$), loss of N-linked glycosylation (prob. score = 0.17, $P = 0.0053$), altered transmembrane protein (prob. score = 0.02, $P = 0.0083$) and a loss of GPI-anchor

amidation. In the gene *nad1* a substitution from F to S (in YEM, at the position 310, ELME000053, ELME000328) changes the ordered interface of the protein (prob. score = 0.24, $P = 0.04$).

Four SNPs were found in tRNAs, of which three were unique to YEM (Met, Cys, Asn) and one to CAL haplotype (Thr). There was also a deletion of a single base pair in tRNA(Asp) in the BRA haplotype. Five SNPs were detected in both long and short rRNAs (*long*: 3 SNPs unique to CAL and 1 in BRA and YEM; *short*: 3 SNPs unique to BRA and 2 to YEM). In addition to genes, the three haplotypes also differ in the two non-coding long intergenic regions recently identified in the mitogenome of seed beetles (see Sayadi et al. 2017).

A comparison of within and between population haplotype divergence

A previous global analysis of *C. maculatus* mtDNA haplotype divergence (Kébé et al. 2017) showed that the total mitochondrial variance is dominated by within-population variance (38.6%) and among-populations-within-region (48.7%) variance, with only a small fraction of the total genetic variance accounted by among-continent variance (12.7%). Nevertheless, to assay sympatric haplotype diversity in *C. maculatus*, we sequenced (forward and reverse) a 625 bp segment of COI from in total 43 isofemale lines from west Africa. Of these, 41 derive from a focal single population collected in a crop field in Lome (Togo) and 2 from a single population named Ofuya (Nigeria). For a description of DNA extraction, primers, PCR-conditions and sequencing, we refer in full to Tuda et al. (2006). In addition, we added sequence data for the same segment of COI from four different reference populations to our analyses. These were the three haplotypes

used in our experimental evolution lines (i.e., California, Yemen, Brazil) as well as the South India (SI) genome reference population (Sayadi et al. 2017, 2019).

Overall, the west African sample showed a very high level of mtDNA haplotype diversity. There were 42 variables sites and 22 out of the 43 haplotypes were unique. Synonymous within-population nucleotide diversity was $\pi_S = 0.041$, which is considerably higher than that between any of the three haplotypes used in our experiment. In addition, there were two non-synonymous polymorphic substitutions in this region ($\pi_N = 0.001$). One involves a substitution from isoleucine to valine, shown by 8 of our 20 unique haplotypes from the Lome population. One out of our four reference sequences (California) also carries this particular non-synonymous substitution and the 8 Lome haplotypes carrying this substitution cluster together with the California haplotype (Figure SI1). The other non-synonymous substitution is from methionine to isoleucine and is carried by a single of our 20 haplotypes from Lome.

A close analysis of this region of COI shows that west African within-population mtDNA variation in *C. maculatus* is comparable to between-population variation (see also Kébé et al. 2017). For example, Sayadi et al. (2017) reported a between-population $\pi_S = 0.062$ for COI and π_S ranged from 0.014 to 0.071 for other protein coding genes of the mitogenome. The relationship among the haplotypes analyzed here is visualized in Figure SI1. These analyses show that two major haplotype groups occur sympatrically in both the Lome and the Ofuya population.

We conclude that although two of the three particular mtDNA haplotypes used in our experimental evolution lines were distinct from those occurring in the Lome population (Figure SI1), the three haplotypes are not more divergent than are haplotypes occurring sympatrically within the Lome population.

SI References

Kebe K, Alvarez N, Tuda M, Arnqvist G, Fox CW, Sembene M, Espindola A (2017) Global phylogeography of the insect pest Callosobruchus maculatus L (Coleoptera: Bruchinae) relates to the history of its main host, Vigna unguiculata L. Journal of Biogeography doi:10.1111/jbi.13052.

Leigh, J. W., & Bryant, D. (2015). PopART: full-feature software for haplotype network construction. Methods in Ecology and Evolution, 6(9), 1110-1116.

Sayadi A, Immonen E, Tellgren-Roth C, Arnqvist G (2017) The evolution of dark matter in the mitogenome of seed beetles. Genome Biology and Evolution 9:2697-2706.

Sayadi A, Martinez Barrio A, Immonen E, Dainat J, Berger D, Tellgren-Roth C, Nystedt B, Arnqvist G (2019) The genomic footprint of sexual conflict. Nature Ecology & Evolution 3:1725–1730.

Tuda, M., Rönn, J., Buranapanichpan, S., Wasano, N. and Arnqvist, G. (2006) Evolutionary diversification of the bean beetle genus Callosobruchus (Coleoptera: Bruchidae): traits associated with stored-product pest status. Molecular Ecology 15:3541–3551.

Supplementary Tables

Table S2. Tests of sex-specific interaction effects between the line type (i.e. mtDNA variation or not), nuclear genetic background, and generation (i.e. 3 or 33 generations in warmer thermal environment), on the lifetime reproductive success.

Fixed effect	*F*	*DF*	*DF res*	*P*
Sex	2.17	1	31.4	0.1505
Nuclear	0.48	2	65.3	0.6183
Generation	0.81	1	31.1	0.3738
Line type	0.07	1	59.2	0.7880
Sex × Nuclear	0.38	2	29.8	0.6896
Sex × Generation	0.61	1	18.6	0.4446
Nuclear × Generation	0.62	2	29.7	0.5442
Line type × Nuclear	0.54	2	54.8	0.5880
Line type × Generation	0.18	1	25.3	0.6734
Line type × Sex	1.12	1	25.7	0.2987
Sex × Nuclear × Generation	0.35	2	19.4	0.7113
Line type × Nuclear × Generation	2.93	2	17.5	0.0802
Line type × Sex × Generation	0.26	1	19.2	0.6176
Line type × Nuclear × Sex	0.48	2	17.6	0.6340
Random effect	*Variance*	*SD*		
Replicate	0.0000	0.0000		
Replicate × Sex	0.0093	0.0966		
Replicate × Generation	0.0134	0.1156		
Replicate × Sex × Generation	0.0354	0.1881		
Residual	0.8552	0.9248		

Table S3. The total numbers of single nucleotide polymorphisms (SNP's) and the numbers of non-synonymous substitutions (N_s) in the mitochondrial protein-coding genes in the three haplotypes.

Gene	Codon number	Haplotype	Codon	Amino acid
nad2	286	CAL	TTA	Leu
		BRA	TTA	Leu
		**YEM**	**ATA**	**Met**
	304	**CA**	**TTA**	**Leu**
		BRA	TCA	Ser
		YEM	TCA	Ser
cox1	292	**CAL**	**GTT**	**Val**
		BRA	ATT	Ile
		YEM	ATT	Ile
	469	**CAL**	**ATA**	**Met**
		BRA	GTA	Val
		YEM	GTT	Val
cox2	27	CAL	GCA	Ala
		**BRA**	**ACA**	**Thr**
		YEM	GCA	Ala
	92	CAL	TAA	Asn
		**BRA**	**TAG**	**Ser**
		YEM	TAA	Asn
cox3	2	CAL	ACA	Thr
		BRA	ACA	Thr
		**YEM**	**GCA**	**Ala**
nad5	86	CAL	ACT	Thr
		**BRA**	**ATT**	**Ile**
		YEM	ACT	Thr
	181	CAL	GAA	Glu
		**BRA**	**AAA**	**Lys**
		YEM	GAA	Glu
	332	CAL	ATA	Met
		**BRA**	**TTA**	**Leu**
		YEM	ATA	Met
	380	**CAL**	**ACC**	**Thr**
		BRA	ATC	Ile
		YEM	ATT	Ile
nad4	164	CAL	ATG	Met
		BRA	ATG	Met
		**YEM**	**ACG**	**Thr**
	248	CAL	ACT	Thr
		**BRA**	**ATT**	**Ile**
		YEM	ACT	Thr
cob	173	CAL	AAC	Asn
		BRA	AAC	Asn
		**YEM**	**AGC**	**Ser**
	296	CAL	GTC	Val
		**BRA**	**ATC**	**Ile**
		YEM	GTT	Val
nad1	18	CAL	ATT	Ile
		BRA	ATT	Ile
		**YEM**	**GTT**	**Val**
	310	CAL	TTT	Phe
		BRA	TTT	Phe
		**YEM**	**TCT**	**Ser**

Table S4. Non-synonymous substitutions in the protein coding mitochondrial genes of the three mtDNA haplotypes.

**Gene**	**Tot SNPs**	**Ns**	**CAL**	**BRA**	**YEM**
nad2	7	2	1	0	6
cox1	38	2	9	8	21
cox2	17	2	2	6	9
atp8	1	0	0	0	1
atp6	10	0	2	2	6
cox3	6	1	0	2	4
nad3	3	0	1	0	2
nad5	20	4	3	5	12
nad4	20	2	4	3	13
nad4L	1	0	0	0	1
nad6	2	0	1	0	1
cob	27	2	3	4	20
nad1	14	2	2	3	9
**Total**	**166**	**17**	**28**	**33**	**105**
% Ns			**17**	**20**	**63**

A)

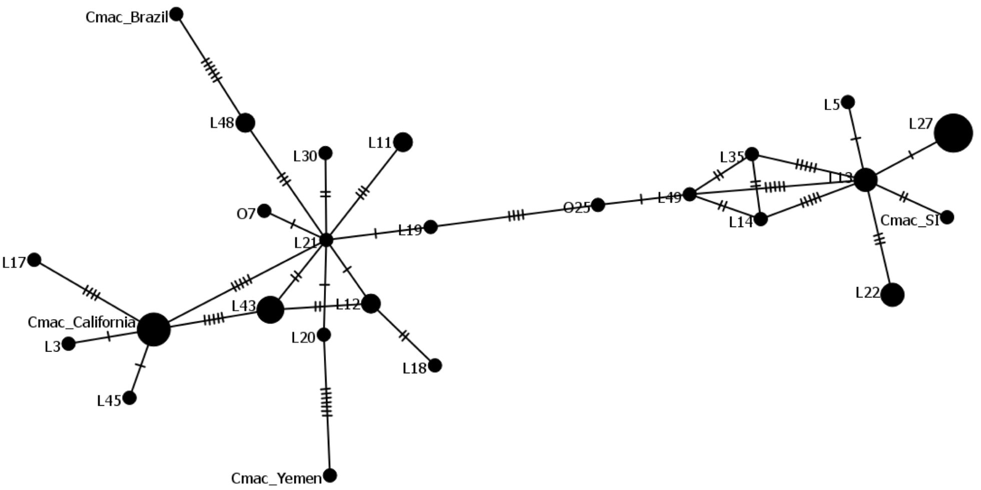

B)

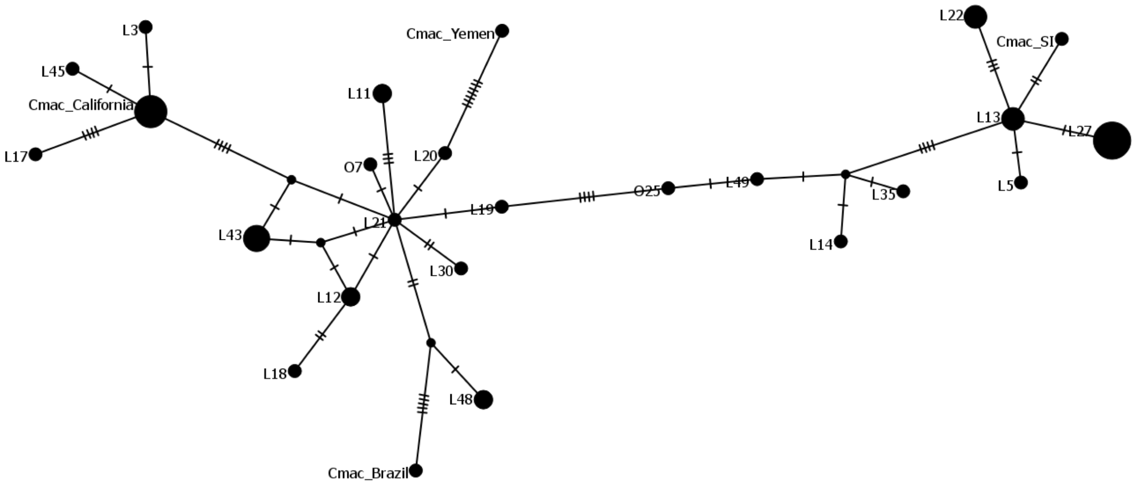

Figure S1. MtDNA haplotype networks based on a 625 bp long segment of COI, showing 42 polymorphic nucleotide positions. Shown here are the Minimum Spanning Network (A) and the Median Joining Network (B) generated in PopART (Leigh & Bryant 2015). Substitutions are shown as hatch marks. Size of dots are proportional to the number of haplotypes shared in each node. There were 26 unique haplotypes, out of a total of 47 haplotypes. L denotes isofemale lines from a single population in Lome and (Togo) O isofemale lines from a population in northern Nigeria. The three haplotypes used in our experiment are denoted by their name, as is the genome reference line SI.

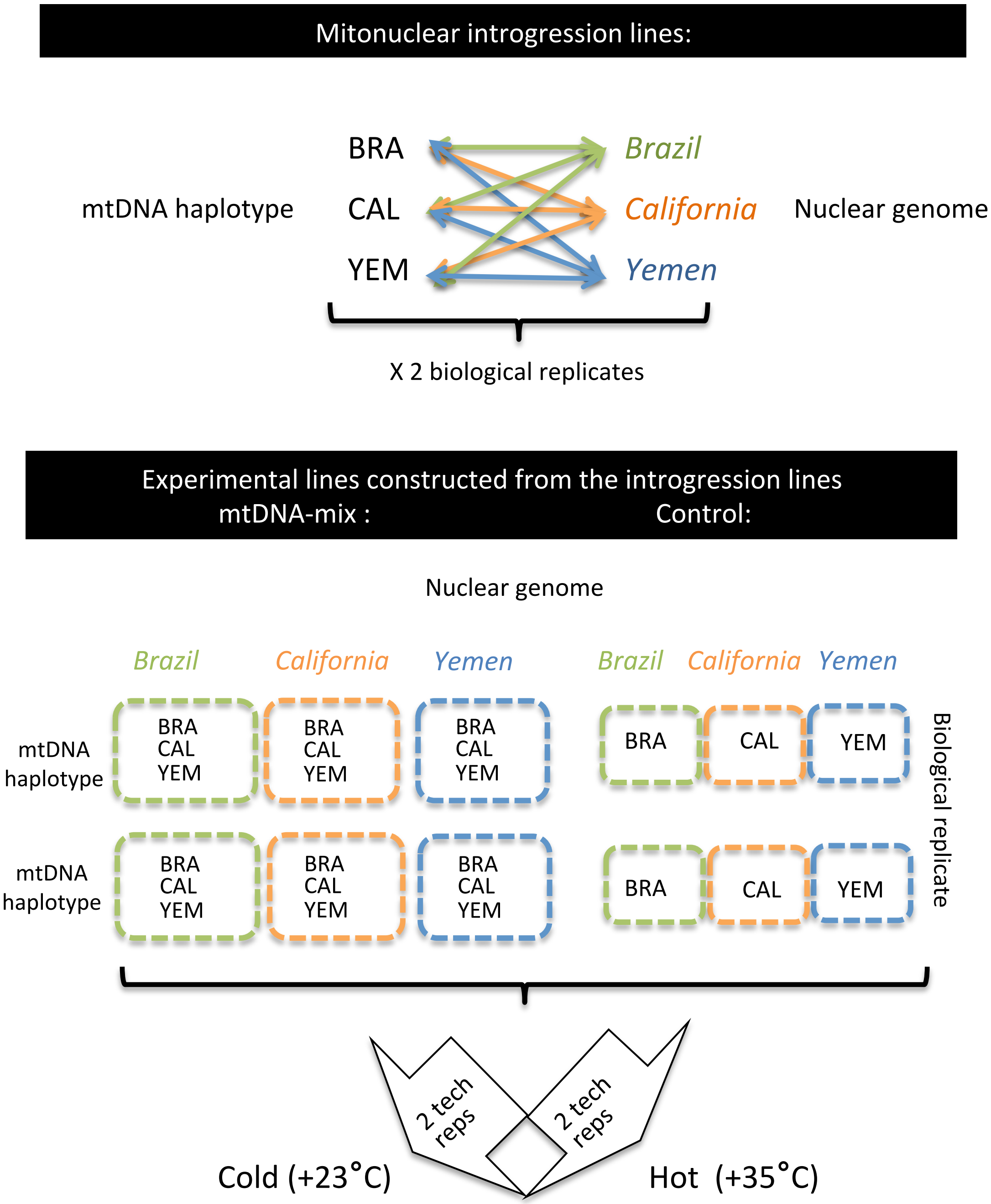

Figure S2. Experimental design. First block shows the orthogonal introgression scheme where each of the three mtDNA haplotypes (BRA, CAL, YEM) where placed alongside two novel and one original nuclear genetic background through repeated backcrossing. The second block shows the scheme for creating the experimental evolution lines. Beetles from the introgression lines sharing the nuclear genetic background (*Brazil, California* or *Yemen*) but harboring different mtDNA haplotype (BRA, CAL or YEM) where placed together in exactly equal proportions (1/3 each) to create new lines with mtDNA variation ("mtDNA-mix"). These lines where then assigned to either colder or warmer thermal conditions. Each mtDNA-mix line was replicated twice using separate introgression lines (i.e. with mtDNA from separate "mitochondrial Eves"), and each of these were split into two replicates per temperature, resulting in a total of eight mtDNA-mix lines per nuclear genetic background. For the reproductive success assays we also used control lines that included the original combination of mtDNA haplotype and nuclear genetic background, created through the same introgression scheme with the same replication.

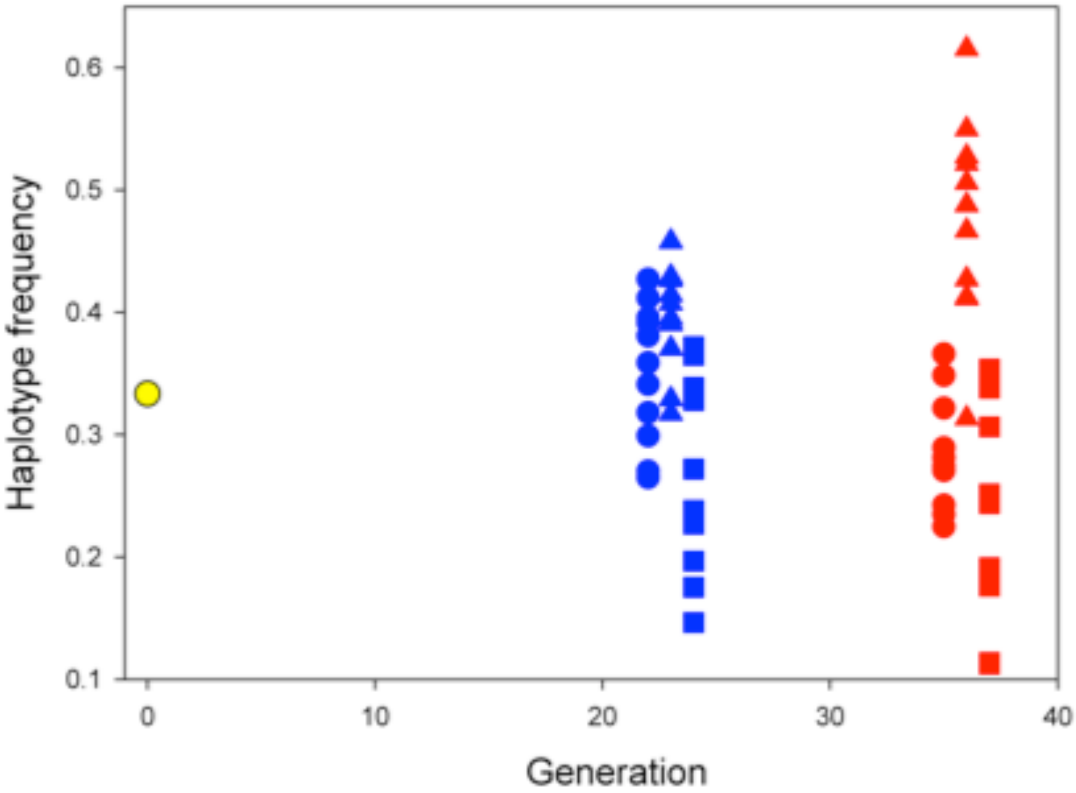

Figure S3. Observed mtDNA haplotype frequency changes in all 23 experimental evolution lines kept under cold (23°C; blue symbols) or hot (35°C; red symbols) conditions. All lines were started with a frequency of 0.33 for each of the three haplotypes at generation 1 (indicated by a yellow circle). The CAL haplotype is denoted by triangles, the YEM by squares and the BRA by circles. Note that haplotype frequencies were estimated at generation 23 (cold) and 36 (warm) but generations are slightly off-set in the figure, to avoid overlap and facilitate visual interpretation.

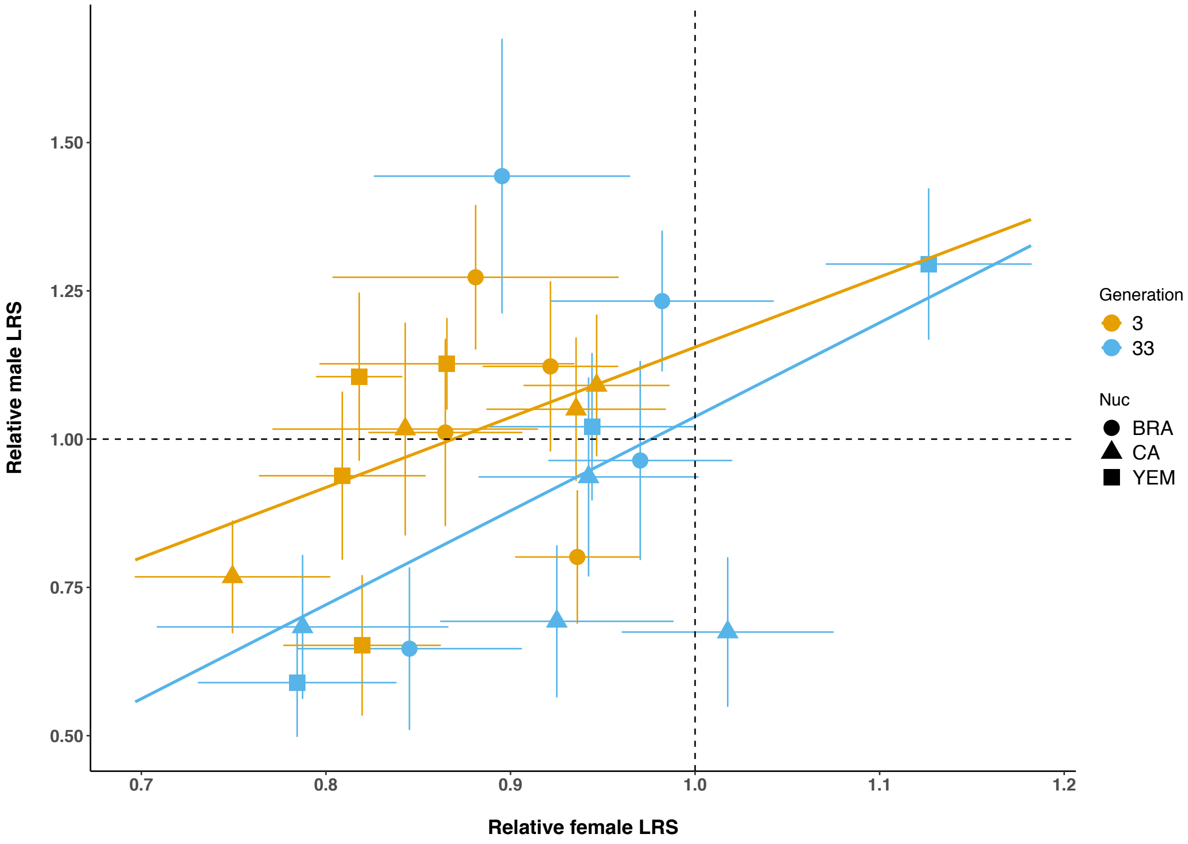

Figure S4. The relative lifetime reproductive success (mean LRS +/- SE, vertical and horizontal lines) of the 'mtDNA-mix' lines harboring mtDNA variation for females and males at the generations 3 and 33. The three different nuclear genetic backgrounds are highlighted with separate symbols. The mean LRS data is plotted relative to the fittest CAL mtDNA haplotype (on its native background). This figure shows how the relative reproductive success of the lines remains sexually concordant across the time points where we verified a significant increase in frequency of the CAL. The horizontal and vertical dashed lines indicate equal fitness in the sexes.

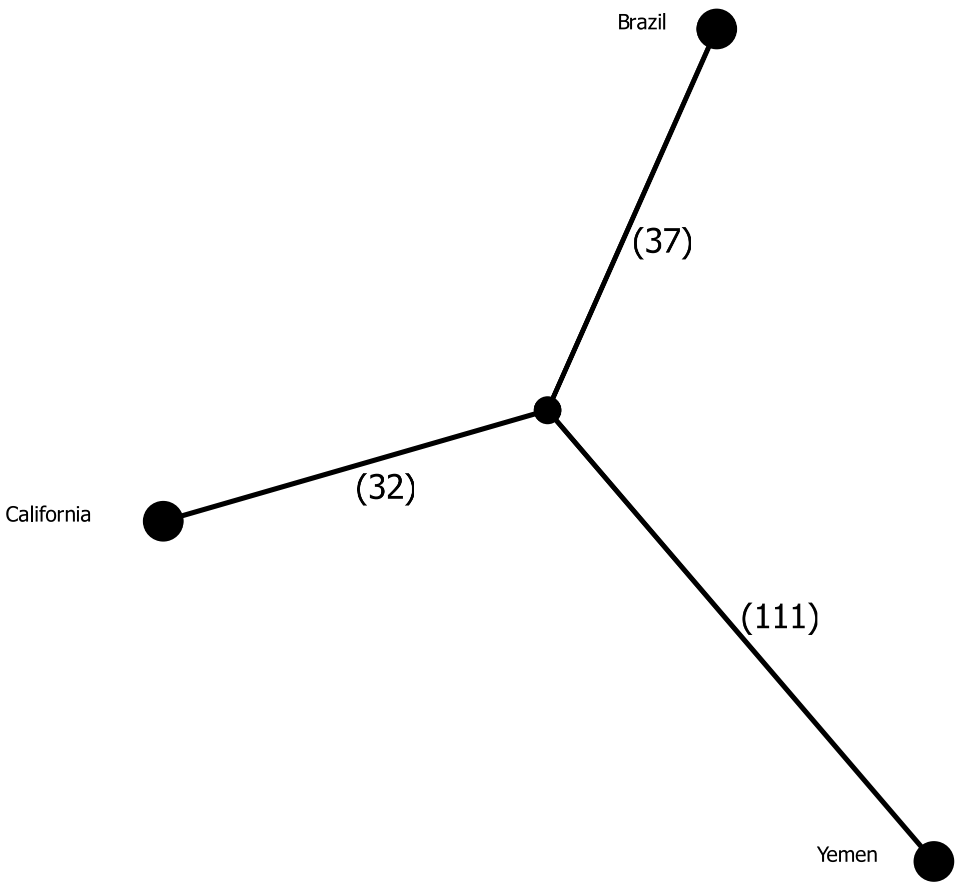

Figure S5. Neighbourhood-Joining based haplotype network of the three haplotypes (closed circles) based on data of all mitochondrial protein coding genes, tRNAs and rRNAs. The number of single nucleotide substitutions is highlighted in parenthesis.
